# Podoplanin (PDPN) affects the invasiveness of thyroid carcinoma cells by inducing ezrin, radixin and moesin (E/R/M) phosphorylation in association with matrix metalloproteinases

**DOI:** 10.1186/s12885-018-5239-z

**Published:** 2019-01-17

**Authors:** Justyna Sikorska, Damian Gaweł, Hanna Domek, Magdalena Rudzińska, Barbara Czarnocka

**Affiliations:** Department of Biochemistry and Molecular Biology, Center of Postgraduate Medical Education, Marymoncka 99/103, 01-813 Warsaw, Poland

**Keywords:** Podoplanin, TPC1, BcPAP, Ezrin/radixin/moesin, Metalloproteinases, Motility, Invasiveness

## Abstract

**Background:**

Podoplanin (PDPN) is a mucin-type transmembrane glycoprotein specific to the lymphatic system. PDPN expression has been found in various human tumors and is considered to be a marker of cancer. We had previously shown that PDPN expression contributes to carcinogenesis in the TPC1 papillary thyroid cancer-derived cell line by enhancing cell migration and invasiveness. The aim of this study was to determine the effect of PDPN down-regulation in another thyroid cancer-derived cell line: BcPAP.

**Methods:**

In order to determine the effects of PDPN on malignant features of BcPAP cells (harboring the *BRAFV600E* mutated allele) and TPC1 cells (carrying the *RET/PTC1* rearrangement), we silenced PDPN in these cells using small interfering RNA (siRNA). The efficacy of PDPN silencing was confirmed by qRT-PCR and Western blotting. Then, we tested the motility and invasiveness of these cells (using scratch test and Transwell assay), their growth capacities F(cell cycle analysis, viability, clonogenic activity) and apoptosis assays), adhesion-independent colony-formation capacities, as well as the effect of PDPN silencing on MMPs expression and activity (zymography).

**Results:**

We found that PDPN-induced cell phenotype depended on the genetic background of thyroid tumor cells. PDPN down-regulation in BcPAP cells was negatively correlated with the migration and invasion, in contrast to TPC1 cells in which PDPN depletion resulted in enhanced migration and invasiveness. Moreover, our results suggest that in BcPAP cells, PDPN may be involved in the epithelial-mesenchymal transition (EMT) through regulating the expression of the ezrin, radixin and moesin (E/R/M) proteins, MMPs 9 and MMP2, remodeling of actin cytoskeleton and cellular protrusions. We also demonstrated that PDPN expression is associated with the MAPK signaling pathway. The inhibition of the MAPK pathway resulted in a decreased PDPN expression, increased E/R/M phosphorylation and reduced cell migration. Additionally, PDPN depleted BcPAP cells treated with inhibitors of MEK1/2 kinases (U0126) or of the BRAF V600E protein (PLX4720) had reduced motility, similar to that previously observed in TPC1 cells after PDPN knock-down.

**Conclusions:**

Altogether, our data suggest that PDPN may play an important role in the control of invasion and migration of papillary thyroid carcinoma cells in association with the E/R/M, MMPs and MAPK kinases.

**Electronic supplementary material:**

The online version of this article (10.1186/s12885-018-5239-z) contains supplementary material, which is available to authorized users.

## Background

Podoplanin (PDPN) is a 38–40 kDa type I mucin-like transmembrane sialoglycoprotein which belongs to the podoplanin family [1–3]. It was initially identified in the kidney as a marker of podocytes [4] but it is also expressed in numerous other human tissues, such as skeletal muscles, heart, placenta, and lung [4–7]. PDPN is a useful marker of lymphatic endothelium and lymphangiogenesis as it is exclusively expressed in the lymphatic endothelial cells (LEC) but not in blood endothelial cells (BEC) [8]. PDPN is also upregulated in a variety of neoplasms, including colorectal tumors [9], squamous cell carcinomas [5, 6, 10, 11], mesothelioma [12], testicular seminoma [13], brain tumors [14–16] and some types of vascular tumors [17, 18] as well as in some papillary thyroid tumors [19]. Interestingly, in IHC analysis of the thyroid tissue specimens podoplnin was not detected in normal thyroid follicular cells or in non-tumorous cells of the peritumoral margin, but only in lymphoid endothelial cells of the lymphatic vessels [19].

PDPN function remains to be fully elucidated. Its role in the separation of the lymphatic and venous system during development by interacting with platelets was shown in several studies [4, 8, 20]. In tumors, PDPN may play an important role as a regulator of tumor cell migration and invasion, thus contributing to cancer progression and conferring poor prognosis [18, 21, 22]. Functional experiments performed in cell lines suggest that PDPN regulates the tumor cell motility. PDPN expression has been shown to promote cell scattering and the extracellular matrix (ECM) degradation in the HaCaT human immortalized keratinocyte cell line [5]. Additionally, it was shown that in-vitro PDPN-mediated invasion depends on the activity of matrix metalloproteinases (MMP) [11, 21] degrading ECM surrounding the tumor [23, 24]. In Madin-Darby Canine kidney cells (MDCK), PDPN has been shown to increase cell motility and induce the epithelial-mesenchymal transition (EMT) by switching the cells’ migration pattern to a fast individualized cell locomotion and increased invasiveness [25]. Furthermore, high ectopic expression of PDPN has been shown to result in enhanced cancer cell migration, lymphangiogenesis and metastasis in the human MCF7 breast carcinoma xenograft model [26]. Moreover, in the same cell line model podoplanin was shown to be involved in collective migration of MCF 7 cells, without inducing EMT [11]. Moreover, Martín-Villar and collaborators showed that PDPN contributes to both directional migration of normal epithelial cells and cells derived from squamous cell carcinomas (SCC) [27].

Microscopy studies revealed that PDPN is concentrated in actin-rich plasma membrane protrusion structures, such as microvilli, ruffles, filopodia, and lamellipodia, where it colocalized with members of the E/R/M protein family. Moreover, ectopic expression of PDPN in murine non-tumorigenic keratinocytic cells was shown to induce of actin cytoskeleton rearrangement and redistribution of ezrin to surface protrusions, as well as to enhance cell motility, suggesting PDPN contribution to the cell migratory potential [28]. Podoplanin could also be considered as an EMT inducer, as the malignant cells expressing PDPN often show a mesenchymal phenotype, that is characteristic feature EMT [29], and the presence of PDPN in lipid rafts is necessary for the recruitment and activation of E/R/M proteins and induction of EMT as well as invadopodia functionality [29–31].

Previously we have demonstrated that PDPN mediates invasiveness in TPC1 cells derived from papillary thyroid carcinoma (PTC), that may suggest the involvement of PDPN in PTC progression. PDPN knock-down in TPC1 cells decreased cellular invasion and reduced cell migration. PDPN is up-regulated in both TPC1 and BcPAP papillary cancer-derived cell lines, however BcPAP cells, harboring the *BRAF V600E* mutation, have higher PDPN expression level. This may suggest that this gain-of-function mutation may be associated with a stronger induction of PDPN expression [19].

Therefore, we extended our analyses to the BcPAP cell line harboring a mutated *BRAF* allele (*BRAF V600E*), and expressing higher level of PDPN than TPC-1 cells. The *BRAF V600E* is a common mutation that plays a crucial role in tumorigenesis and progression of PTC [32–34]. Although signaling pathways activated by *BRAFV600E* and *RET/PTC1* overlap, the tumors associated with each of these two alterations have unique phenotypic features, suggesting that they also may have different tumor biology [35–39].

Therefore, in the current study, we compared the function of PDPN expressed in PTC derived cell lines with different genetic background on the modulation of cell motility, migration and invasion associated with tumor progression. We demonstrate that PDPN knock-down either promote or suppress metastatic potential of thyroid cancer cell depending on the genetic background. Overall, our results suggest and support the role of PDPN in thyroid tumorigenesis.

## Methods

### Cell lines and cell culture

We used two thyroid cell lines derived from papillary thyroid carcinoma: BcPAP (German Collection of Microorganisms and Cell Cultures) which was previously tested and authenticated by DNA analysis, and TPC1 (established by Dr. Nobuo Sato) [40] and kindly provided by Dr. M. Santoro, The University of Naples Federico II, Italy)**.** These cell lines differ in their genetic background: BcPAP cells carry the *V600E* mutation in the *BRAF* gene, while TPC1 cells harbor the *RET/PTC1* rearrangement. Both cell lines were cultured in RPMI-1640 medium (Lonza, Switzerland) supplemented with 10% fetal bovine serum (FBS; Thermo Fisher Scientific, USA), in humidified 5% CO2 atmosphere at 37 °C. Viable cells were enumerated on the EVA Automatic Cell counter (Nano EnTek, Korea) following Trypane Blue staining. Both cell lines were tested for mycoplasma contamination on a regular basis.

### PDPN silencing with small interfering RNA (siRNA)

BcPAP and TPC1 cells were transfected with siRNA targeting human PDPN (Thermo Fisher Scientific, Ambion, USA) and a universal negative control siRNA (MISSION® siRNA Universal Negative Control #1, Sigma-Aldrich, USA) as described previously [19]. The efficiency of the PDPN knock-down was assessed by quantitative PCR of reverse-transcribed RNA (RT-qPCR), Western blotting and immunofluorescence (IF) 48 h after transfection. The experiment was repeated at least three times.

### RNA isolation and RT-qPCR

Total RNA was isolated from thyroid cancer cell lines 48 h after transfection using the GeneMATRIX Universal RNA Purification Kit (EURx, Poland) in accordance with the manufacturer’s instructions. Total RNA (500 ng) was reverse-transcribed using the Takara Reverse Transcription Kit (Takara, Japan). Then, qPCR reactions were performed as described previously [19], the mixture containing a Maxima SYBR Green/Fluorescein qPCR Master Mix (Thermo Scientific, Canada) and specific oligonucleotide primers (Table 1).

**Table 1:** Sequences of used qPCR primers

Gene name	Forward primer sequence	Reverse primer sequence
PDPN	5’-CGAAGATGATGTGGTGACTC-3′	5’-CGATGCGAATGCCTGTTAC-3′
EZR	5′- TCTTCGCTGCTGCTGGATAG-3′	5’-GGTGGTAACTCGGACATTGATTG-3′
MSN	5’-TGAGGCTGTGGAGTGGCAGC-3′	5’-CTAGAGGCTGGGTGCCCATT-3′
RDX	5’-GGCAACACAAAGCTTTTGCA-3′	5’-ATATATGCAAAATAACAGCTC-3′
MMP1	5’-CTGGCCACAACTGCCAAATG-3′	5’-CTGTCCCTGAACAGCCCAGTACTTA-3′
MMP2	5’-CAGGGAATGAGTACTGGGTCTATT-3′	5’-ACTCCAGTTAAAGGCAGCATCTAC-3′
MMP3	5’-GAAATGAGGTACGAGCTGGATACC-3’	5’-ATGGCTGCATCGATTTTCCT-3’
MMP7	5’-GCCTACCTATAACTGGAATG-3’	5’-AGCCTTTGACACTAATCG-3’
MMP9	5’-GCACGACGTCTTCCAGTACC-3’	5’-CAGGATGTCATAGGTCACGTAGC-3’
MMP13	5’-TCCCAGGAATTGGTGATAAAGTAGA-3’	5’CTGGCATGACGCGAACAATA-3’
SNAI1	5’-CCCAATCGGAAGCCTAACTA-3’	5’-CAGGACAGAGTCCCAGATGAG-3’
TWIST1	5’-CATCGACTTCCTCTACCAGGTC-3’	5’-TCCATTTTCTCCTTCTCTGGAA-3’
RHOA	5’-TGCTTGCTCATAGTCTTCAG-3’	5’-CACATCAGTATAACATCGGTATC-3’
PTK2	5’-GGTGCAATGGAGCGAGTATT-3’	5’-GCCAGTGAACCTCCTCTGA-3’
PXN	5’-ACGTCTACAGCTTCCCCAACAA-3’	5’-AGCAGGCGGTCGAGTTCA-3’
VIM	5’-GACAATGCGTCTCTGGCACGTCTT-3’	5-TCCTCCGCCTCCTGCAGGTTCTT-3’
18S rRNA	5’-CCAGTAAGTGCGGGGTCATAAG-3’	5’-CCATCCAATCGGTAGTAGCG-3’

Amplification, data acquisition and data analysis were carried out using the iQ5 Real-Time PCR Detection System and software (Bio-Rad, USA).

### Protein extraction, polyacrylamide gel electrophoresis (PAGE) and Western blotting

For protein extraction, the cells were detached from culture vessels 48 h after transfection, and lysed as follows. The cell pellets were re-suspended in the RIPA lysis buffer (150 mM sodium chloride; 1.0% NP-40; 0.5% sodium deoxycholate; 0.1% SDS; 50 mM Tris, pH 8.0) supplemented with 1% protease and phosphatase inhibitor cocktail (Roche, Switzerland). Next, 30μg of proteins were separated by electrophoresis in 10% SDS-PAGE gels and then electro-transferred onto nitrocellulose membranes (Bio-Rad, USA). After blocking the non-specific sites with 5% BSA or 5% non-fat dry milk in TBS-T (Tris-buffered saline, pH 8.0-Tween 20 0,05%), membranes were probed with primary antibodies (Table 2) by overnight incubation at 4 °C, followed by incubation with the appropriate HRP-conjugated secondary antibodies (Jackson ImmunoResearch Laboratories, USA; Table 2). Finally, specific protein signals were detected using the SuperSignal West Pico Chemiluminescent Substrate or SuperSignal West Dura Extended Duration Substrate (ThermoFisher Scientific, USA) on ChemiDoc XRS^+^ Imaging System (BioRad, USA) and quantified using the Quantity One software (BioRad, USA). As a loading control, the membranes were re-probed with a monoclonal anti-β-actin antibody (Sigma-Aldrich, USA) following the same protocol.

**Table 2:** Primary antibodies used by Western blotting and immunofluorescence studies

Targeted protein	Other characteristics	Antibody name (manufacturer)
podoplanin	mouse mAb	D2–40 (Biorad, AbD Serotec, USA)
phospho-ERM	rabbit mAb	48G2 (Cell Signaling, USA)
phospho-p44/42 MAPK (Erk1/2)	rabbit mAb	20G11 (Cell Signaling, USA)
FAK	rabbit Ab	Phospho FAK (Tyr397) (Cell Signaling, USA)
CD44	mouse mAb	156-3C11 (Cell Signaling, USA)
paxillin	rabbit Ab	H-114 (Santa Cruz, USA)
vimentin	mouse mAb	Vim 3B4 (Abcam, UK)
snail	goat Ab	ab53519, (Abcam, UK)
twist	rabbit Ab	ab50581, (Abcam, UK)
RhoA	rabbit mAb	67B9 (Cell Signaling, USA)

*mAb*: monoclonal antibody

### Immunofluorescent staining

Forty-eight hours following transfection, cells were seeded on uncoated glass coverslips in 24-well plates at 1 × 10^5^ cells/ml and incubated for 72 h. Then, they were fixed in 4% PFA/PBS for 15 min and permeabilized in 0.25% Triton®X-100 for 10 min. After blocking the non-specific sites in 2% BSA/PBS-T (phosphate buffered saline, pH 7.3, 0.05% Tween 20), cells were incubated with the following primary antibodies at 4 °C overnight: anti-phospho-ERM rabbit mAb (48G2, Cell Signaling, USA), anti-MMP2 mouse mAb (6E3F8, Abcam, UK), anti-MMP9 mouse mAb (5G3, Abcam, UK), and Phalloidin-FITC (Sigma-Aldrich, USA). Next, after several washes with PBST, the cells were incubated for one hour in the dark with the secondary antibodies: anti-mouse DyLight™549-conjugated, or goat anti-rabbit TRITC- or FITC-conjugated antibodies (Jackson ImmunoResearch Laboratories, USA). The cells were then counterstained with nuclear dye DAPI and visualized under a fluorescent and/or confocal microscope (AxioObserver D1 and AxioObserver Z1, Zeiss, Germany) using oil-immersion lenses. PDPN protein immunostaining was performed as described elsewhere [19].

### Cell migration and invasion assays

Cell migration and invasion activity assays were performed as previously described [19] using Falcon® Permeable Support for-24 Well Plate (8.0 μm pore size) and Corning® BioCoat™ Matrigel® Invasion Chambers (8.0 μm) (Corning, USA). The cells that migrated were stained with a Diff-Quik Kit (Medion Diagnostics, Switzerland) and counted at a 40x magnification under the Olympus BX41 microscope. Each experiment was performed in triplicate and repeated three times.

### In vitro wound healing motility assay

The cell motility assay was performed as described elsewhere [19]. The width of the wound was measured immediately after wounding (time 0 h) and at 6-h intervals during 24 h. Wound images were taken using a phase contrast microscope (AxioObserver D1, Zeiss, Germany) and measured in the ImageJ platform. The migration ability of the cells was then calculated by dividing the values by two and subtracting the results from the initial half-wound width as described by others [41].

### Colony formation assay

After PDPN silencing or inhibitor treatment experiments, the TPC1 and BcPAP cells were counted using the EVE automatic cell counter (NanoEnTek, Korea). Then, 500 cells were seeded in one 100 mm Petri dish (three replicates for each experiment) and incubated in a 5% CO_2_ humidified environment at 37 °C for 7–12 days, depending on the cell line. Next, the cells were fixed with 10% PFA for 25 min, washed twice with water, and stained with crystal violet (0.5% *w*/*v*) for 15 min. Digital images of the dried colonies were obtained using a scanning device and visible clones were counted using ImageJ.

### Anchorage-independent cell growth assay

The soft agar colony formation assay was performed in 6-well culture plates. First, 3 ml of 0.64% bottom agar (Roth, Germany) diluted in 10% FBS-RPMI medium were added to each well and the plates were set aside for the agar to solidify. Meanwhile, the top agar layer was prepared by mixing the appropriate number of cells with the complete culture medium and 1.2% agar solution. The final mixture for one well contained 2 ml of 0.3% agar-medium solution with 0.5 × 10^4^ TPC1 cells or 1 × 10^4^ BcPAP cells. Next, the dishes containing two agar layers and cells were incubated at 37 °C for two weeks. Fresh culture medium (10% FBS-RPMI) was added after three days and then changed every three days. After two weeks, cell colonies were counted under a light microscope (AxioObserver D1, Zeiss, Germany) at a 10x and 40x magnification.

### Cell adhesion assay

The assay was performed using the colorimetric ECM Cell Adhesion Array Kit (Merck Millipore, Germany) and also 96-well plates coated by ourselves. The ECM Array microtiter plates, pre-coated with different extracellular matrix proteins (Purified human Collagen I, Collagen II, Collagen IV, Fibronectin, Laminin, Tenascin, Vitronectin), were used according to the manufacturer’s instructions. Independently, 96-well culture microplates (Corning, USA) were coated with fibronectin (20 μg/ml) or laminin-1 (10 μg/ml), and incubated at 4 °C overnight. Non-specific binding sites on the plates were blocked by a one-hour incubation with 0.5% BSA in the RPMI medium at 37 °C in a CO_2_ incubator. Next, the cells were counted and re-suspended in the culture medium to final concentration. Then, 50 μl of cell suspension of 4 × 10^5^ cells/ml were seeded in the wells (in triplicates) and allowed to adhere for 30–60 min at 37 °C. After that, unattached cells were removed by washing, and adherent cells were fixed with 4% PFA for 15 min and then stained with crystal violet solution (Sigma Aldrich, USA) for 10 min. After extensive washing, the dye was extracted using 2% SDS and quantified by spectrophotometry at 550 nm using a microplate reader (Multiskan RC, Labsystems, Thermo, Finland).

### Cell viability assay

The number of proliferating cells was determined by measuring the amount of 5-bromo-2′-doexyuridine (BrdU) incorporated into newly synthetized DNA using the BrdU Cell Proliferation Assay (Merck Millipore, USA). Forty eight hours after transfection with siRNA, the cells were seeded in 96-well plates (5 replicates) and BrdU was added to each well. Next, the cells were incubated at 37 °C for 17 h in a 5% CO_2_ incubator and then treated following the manufacturer’s instructions. Absorbance was measured at the test wavelength of 450 nm and the reference wavelength of 595 nm in the Labsystems Multiscan RC microplate reader (Thermo, Finland).

### Apoptosis assay

Apoptosis was analyzed using the Annexin V-FITC Apoptosis Detection Kit (Abcam, UK) according to the manufacturer’s protocol. Briefly, the cells were harvested, washed with PBS and incubated with FITC-Annexin V and propidium iodide for 5 min at room temperature. Afterwards, the labeled cells were analyzed by flow cytometry using BD FACSCanto™ II system (BD Biosciences, USA). The experiment was performed three times.

### Cell cycle analysis

To distinguish cells in different phases of the cell cycle, propidium iodide (PI) staining was performed. The cells were harvested 48 h after transfection, washed with PBS and fixed with 70% ethanol at − 20 °C overnight. Then, the cells were washed twice, permeabilized with 0.1% NP-40 for 15 min and stained with 5 μg/ml PI solution (Sigma-Aldrich, USA). Stained cells were analyzed by flow cytometry (FACSCantoII, BD Biosciences, USA). The experiment was repeated three times.

### Gelatin and collagen zymography

For MMP activity measurement, cells 48 h after PDPN silencing the cells were washed with PBS and grown in serum-free medium at 37 °C in a humidified 5% CO_2_ atmosphere. After 24 h, the culture supernatant was collected and immediately stored at − 70 °C until further processing. Next, non-denatured conditioned medium samples were resolved using non-reducing SDS-PAGE, with gels containing 2 mg/ml gelatin or 0.5 mg/ml collagen type I (BD Biosciences, Bedford, MA, USA). Then, the gels were washed twice in 2.5% Triton X-100 in H_2_O for 30 min and incubated in a developing buffer (50 mM Tris-HCl, pH 7.5, 10 mM CaCl_2_, 1 μM ZnCl_2_, 1% Triton X-100, 0.02% NaN_3_) for 3.5 days. Afterwards, they were staining with 0.5% Coomassie Brilliant Blue R250 in 30% ethanol, 10% acetic acid for 30 min, and then destained with 30% ethanol and 10% acetic acid. Finally, gel images were captured on the ChemiDoc XRS+ Imaging System (BioRad, Hercules, CA, USA). The bands formed by MMP enzymatic activity were quantified using the Quantity One software (BioRad, Hercules, CA, USA).

### Inhibition of the MAPK signaling pathway

To inhibit the MAPK signaling pathway in the studied cell lines we used two selective inhibitors: U0126 and PLX4720. U0126 (1,4-diamino-2,3-dicyano-1,4-bis[2-aminophenylthio] butadiene; Cell Signaling, USA) is a highly selective inhibitor of MEK 1 and MEK 2, and thus efficiently down-regulates the MAPK signaling pathway. PLX4720 (Abcam, UK), blocks the activation of the MAPK signaling by inhibiting ERK phosphorylation in cell lines carrying the *BRAF V600E* mutation but not in cells with wild-type *BRAF*. The BcPAP and TPC1 cells were treated with 50 uM of U0126 or 10 uM of PLX4720, both diluted in DMSO, for 24 h. Cells treated with DMSO only were used as a negative control. The inhibition of MEK1/2 or ERK was confirmed by Western blotting.

### Data analysis

Statistical data analyses and graphs were made with the Prism 6 software (GraphPad, USA). Statistical significance was determined using the nonparametric Mann-Whitney U test and paired t-test, with the *P* value below 0.05 being considered as indicative of a statistical significance. All results are presented as means with standard errors of the mean (SEMs) from at least three independent experiments.

## Results

### Podoplanin silencing in papillary thyroid cancer cell lines

**Fig. 1:**
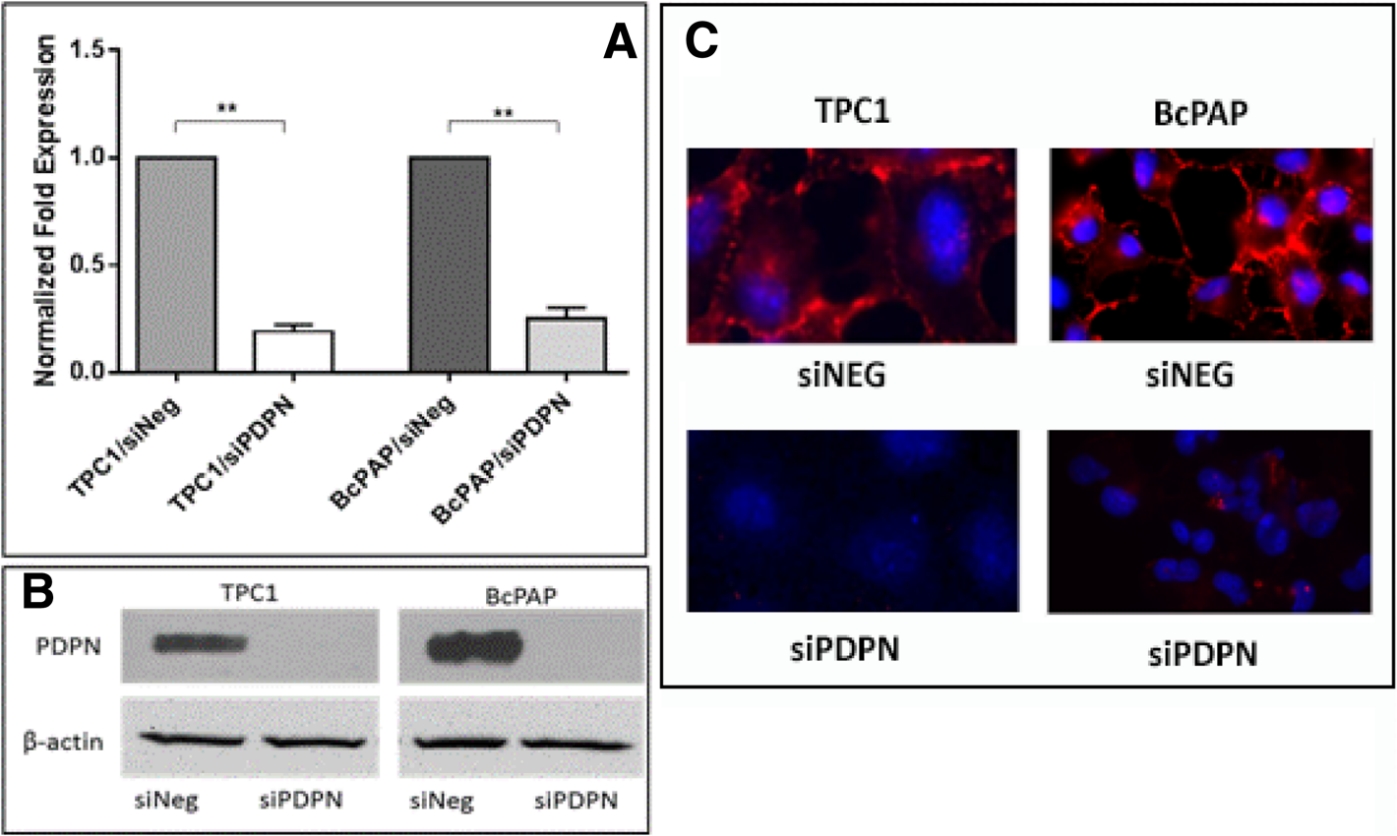
Downregulation of podoplanin expression in BcPAP thyroid cancer cells following transfection with PDPN-specific siRNA. **a.** RT-qPCR analysis of PDPN mRNA levels in BcPAP cells 48 h after transfection with 30 nM siRNA specific for PDPN (siPDPN) or negative control siRNA (siNeg). The results were normalized to the 18S rRNA levels. The bars represent the average fold-change in PDPN transcript levels in cells transfected with siPDPN compared to control cells. The results are representative of four independent experiments. Data are presented as means ± standard errors of the mean (SEM), **: *P* < 0.01. **b.** Podoplanin protein levels in BcPAP cells 48 h after transfection with siPDPN or control siNeg by Western blot. β-actin was used as loading control **c.** Immunofluorescent staining of podoplanin in BcPAP cells transfected with siPDPN or control siNeg. Cells were stained with the anti-PDPN monoclonal antibody D2–40 followed by the DyLight549-conjugated secondary antibody (red), and counterstained with DAPI (blue). Magnification: 630x

In order to assess the role of PDPN in the biology of papillary thyroid cancer, we silenced the expression of PDPN in two papillary thyroid cancer cell lines: TPC1 and BcPAP by transfection with targeted siRNA (siPDPN). Negative control cells were transfected with negative siRNA (siNeg). RT-qPCR analyses demonstrated that siPDPN efficiently decreases PDPN levels 48 h after transfection in both thyroid cancer cell lines. We observed a four-fold reduction in the PDPN mRNA expression in BcPAP cell line (1.00 versus 0.25 in siNeg-transfected cells, *P* < 0.01) and an over five-fold reduction in TPC1 cells (1.00 versus 0.19, *P* < 0.01; Fig. 1a). Although the cells retained low levels of PDPN mRNA which were detectable by qRT-PCR following transfection, PDPN protein levels were below the detection thresholds of Western blot and immunofluorescence analyses in both BcPAP and TPC1 cell lines (Fig. 1b-c).

### The effect of podoplanin silencing on the motility, invasiveness, adhesion and proliferation of papillary thyroid cancer cells

**Fig. 2:**
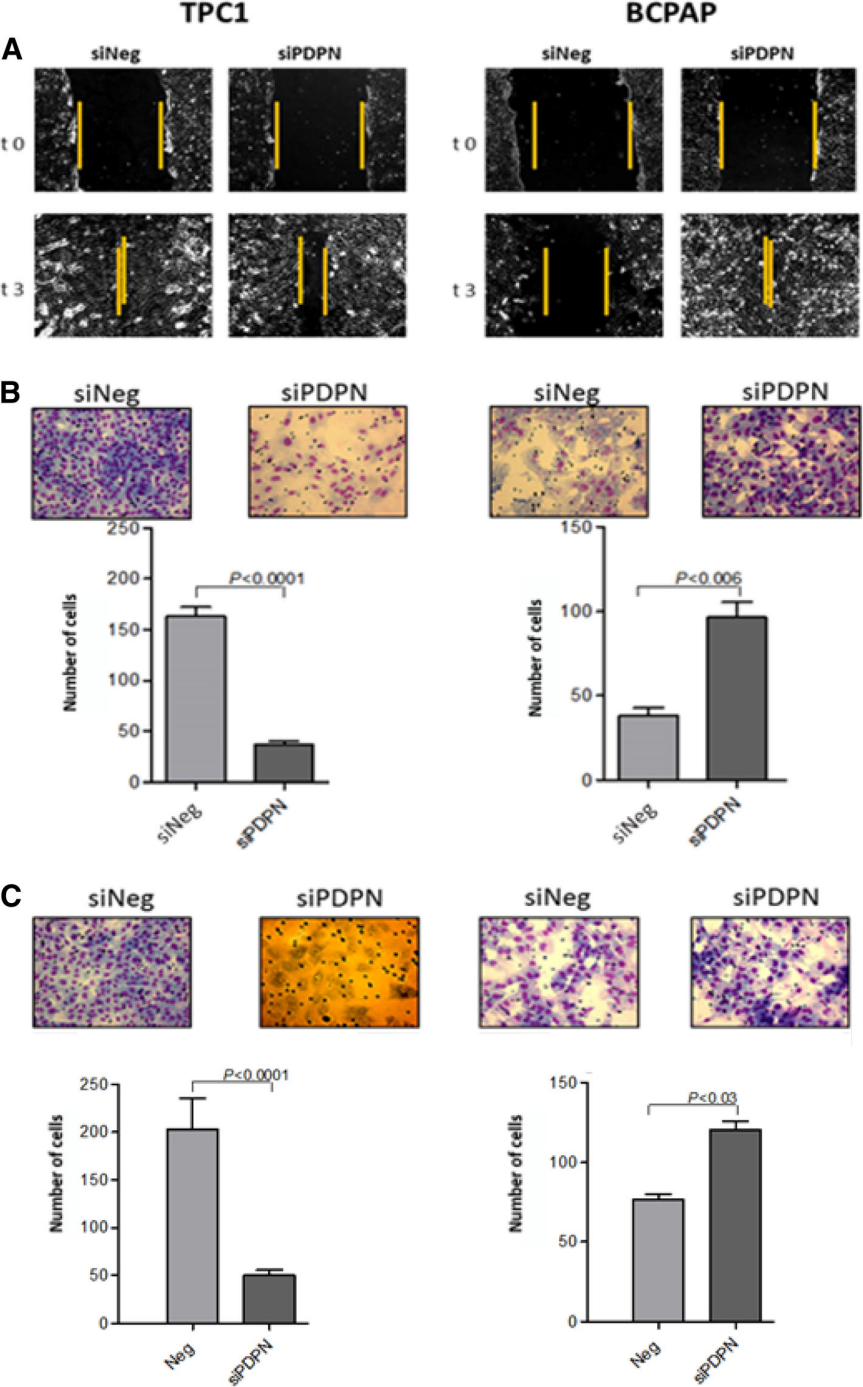
Podoplanin silencing reduces motility, migratory capacities and invasiveness of BcPAP and TPC1 cells. **a.** Wound healing motility assay. Representative light microscope images showing healing of wounds in monolayers of TPC1 and BcPAP cells transfected with siPDPN or control siNeg Magnification: 200x. **b.** Chamber migration assay. siPDPN or siNeg-transfected TPC1 and BcPAP cells were seeded in 8-μm Boyden insert chambers, with the lower reservoir filled with medium supplemented with 10% FBS as a chemoattractant. After 24 h, cells that had passed through the 8-μm membrane pores were fixed, stained with Diff-Quik and photographed at a 40x magnification.. Data are presented as means ± standard errors of the mean (SEM) of at least three independet experiments. **c.** Matrigel invasion assay. siPDPN- or control siNeg-transfected TPC1 and BcPAP cells were seeded in 8-μm Matrigel Invasion Chambers, with the lower reservoir filled with the culture medium supplemented with 10% FBS as a chemoattractant. After 24 h, cells that had passed through the 8-μm pores in the membrane were fixed with, stained with Diff-Quik Kit, and photographed at 40x magnification. The relative invasiveness of transfected cells is presented in graph form. Data are presented as the mean ± SEM of at least three separate experiments

Since the migration and invasion of cancer cells are crucial factors responsible for cancer progression, we first examined whether PDPN may impact the ability of cells to migrate. To this end, we performed classical wound healing assay and a chamber migration assay. Wound healing assays showed that TPC1 and BcPAP cells had different migration potentials: the PDPN-depleted BcPAP cells migrated faster than PDPN-depleted TPC1 cells (Fig. 2a). Similar results were obtained with the chamber migration test. The PDPN-depleted BcPAP cells migrated faster than siNeg-transfected control cells (98 and 36 cells/24 h, respectively), whereas TPC1 cells transfected with siPDPN migrated slower than control TPC1 cells (36 versus 164 cells/24 h; Fig. 2b).

First, we tested the motility and migratory capacities of the cells by wound healing and chamber migration assays. We observed that in the BcPAP cell line the depletion of PDPN expression promotes cells migration, in contrast to the pro-metastatic role of PDPN in TPC1 cells. The effect of PDPN silencing on the motility of thyroid cancer cells (TPC1 and BcPAP) was investigated using a classical wound healing assay and a chamber migration assay. Wound healing assays showed that TPC1 and BcPAP cells had different migration potentials: the PDPN depleted BcPAP cells migrated faster than PDPN depleted TPC1 cells (Fig. 2a). Similar results were obtained by the chamber migration test. We found that BcPAP cells treated with siPDPN migrated faster than siNeg-transfected control cells (97.6 and 36 cells/24 h, respectively), whereas TPC1 cells transfected with siPDPN migrated slower than control TPC1 cells (36 versus 164 cells/24 h; Fig. 2b).

Next, we analyzed the effect of reduced PDPN expression on the invasive potential of the cells by Matrigel invasion assay. We found that invasiveness of PDPN depleted BcPAP cells was enhanced compared to control siNeg-transfected BcPAP cells (121.9 and 76.8 cells/24 h, respectively). This was in contrast to the TPC1 cell line in which the presence of PDPN promoted cell invasion (52 cells/24 h for the PDPN depleted TPC1 cells versus 200 cells/24 h in siNeg-TPC1; Fig. 2c).

**Fig. 3:**
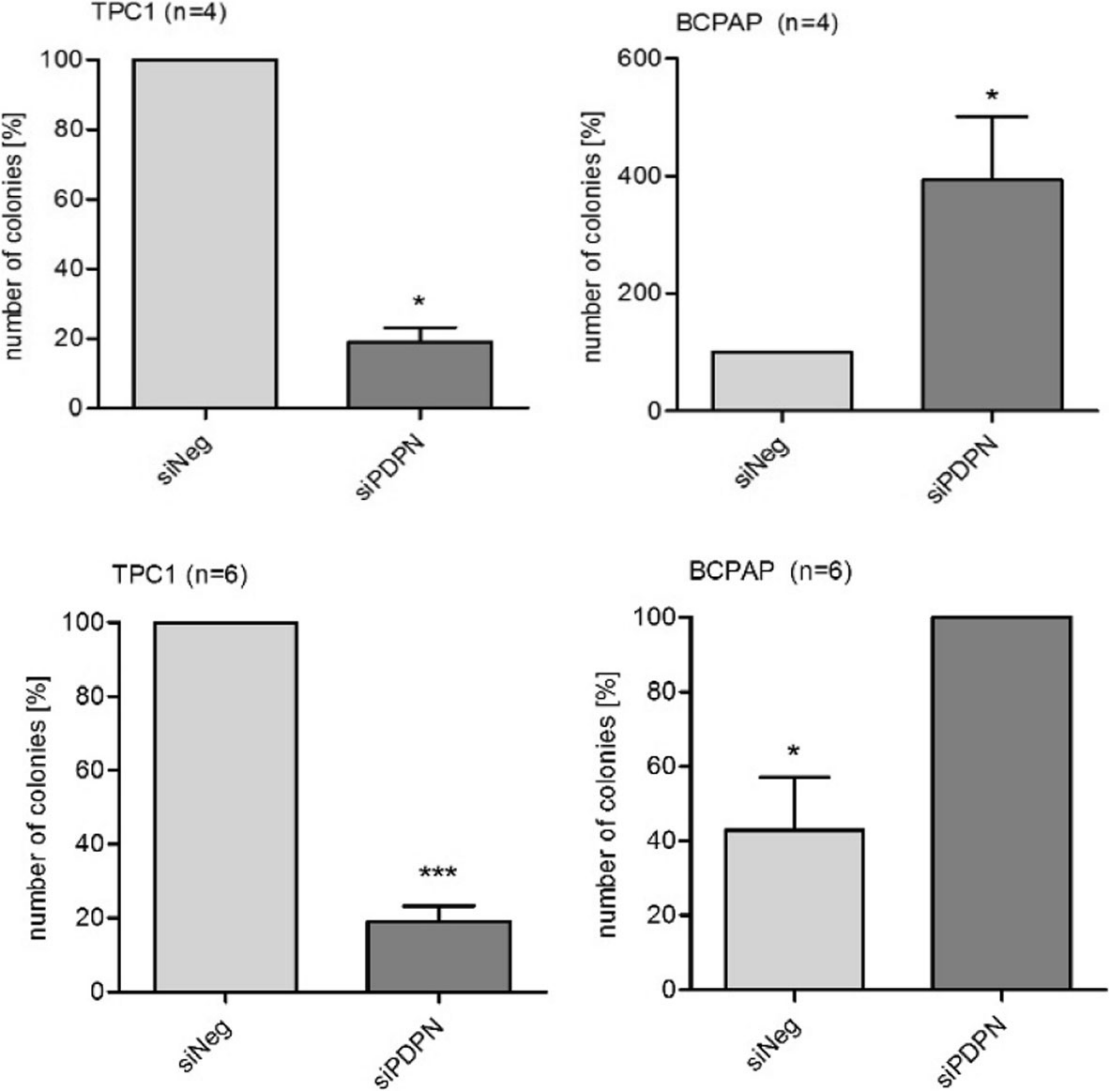
Soft agar (upper panel) and colony formation assay (lower panel**).** To analyze colony formation and anchorage-independent growth of TPC1 and BcPAP cells after PDPN silencing, the cells were seeded into 100 mm Petri dishes or 6-well culture plates containing soft agar. Colonies were stained with crystal violet and counted under a white light microscope at a 10x and 40x magnification. Data are presented as means ± standard errors of the mean (SEM) of results from at least three independent experiments, * < P, 0.05, .*** < P,0.001

Furthermore, we analyzed whether PDPN silencing affects the colony formation and anchorage-independent growth of TPC1 and BcPAP cells. We found that reduced PDPN expression levels in BcPAP cell line increased its capacity to grow both on and independently of the substrate, in contrast to the phenotype observed for the PDPN depleted TPC1 cells (Fig. 3).

Finally we determined whether PDPN depletion influences the adhesion, viability/proliferation, cell cycle and apoptosis in BcPAP and TPC1 cells. We did not detect a consistent effect on cells adhesiveness, G_1_/S cell cycle arrest or viability (proliferation and apoptosis) of both studied thyroid cancer cell lines as shown by adhesion array as well as cell proliferation and viability assays (Additional file 1).

### Podoplanin knock-down enhances phosphorylation of E/R/M proteins

ERM proteins act as crosslinkers between the cell membrane and actin cytoskeleton. They are also involved in regulating such cellular processes as reorganization of actin cytoskeleton, membrane dynamics, cell adhesion, and migration. Therefore, we analyzed the expression of E/R/M transcript and protein levels, and their phosphorylation status in BcPAP and TPC1 cells transfected with siPDPN and control siNeg RNA.

**Fig. 4:**
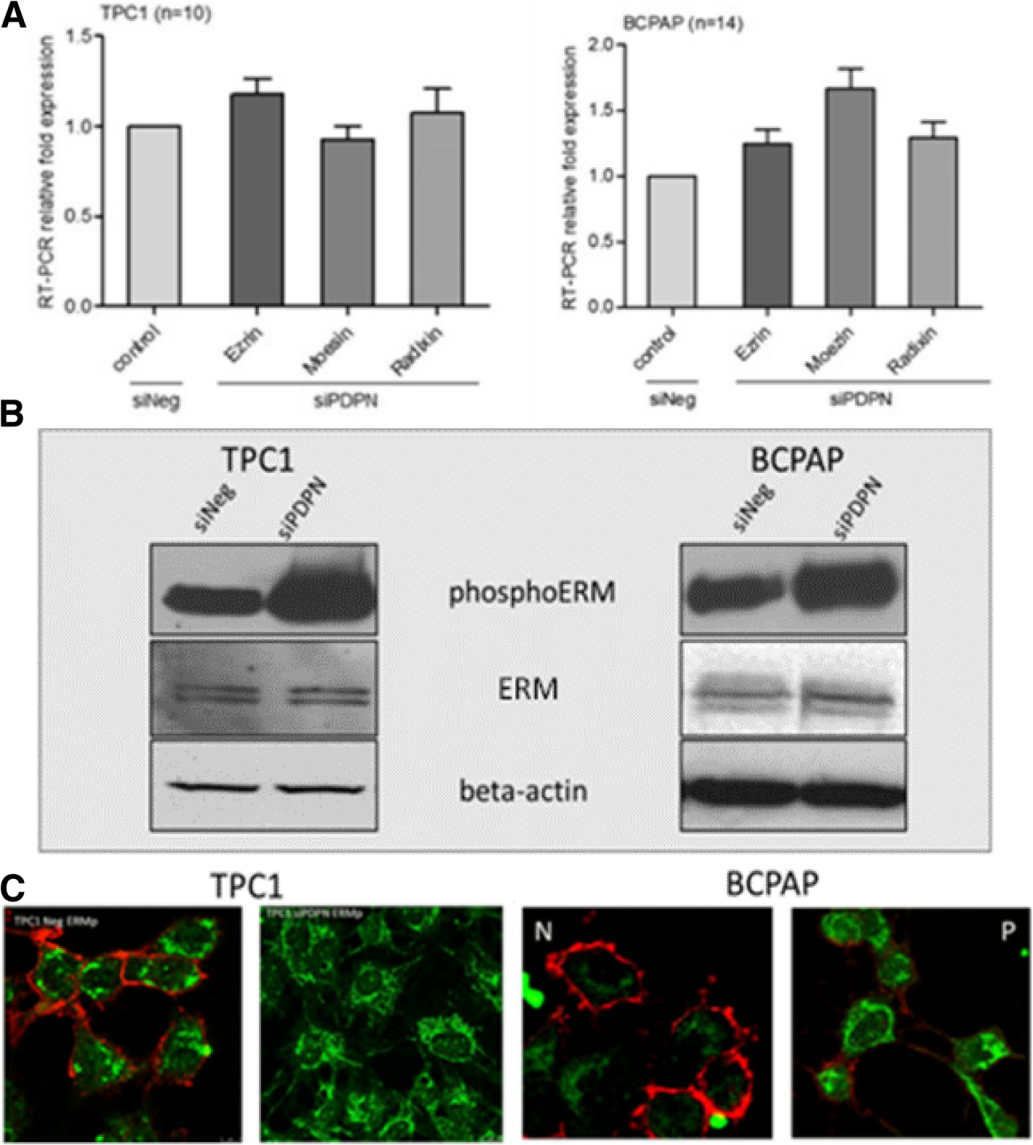
Podoplanin silencing enhances phosphorylation of Ezrin, Radixin and Moesin (E/R/M) proteins in TPC1 and BcPAP thyroid cancer cells. **a.** RT-qPCR analysis of E/R/M mRNA levels 48 h after transfection with siPDPN or control siNeg. The results were normalized to the 18S rRNA levels. The bars represent the average fold change in transcript levels in cells transfected with siPDPN compared to siNeg-transfected control cells. Data are presented as means ± standard errors of the mean (SEM) of results from at least three independent experiments. **b.** Western blot analysis of phospho-E/R/M protein levels 48 h after transfection. Total protein extracts were separated by SDS-PAGE, transferred onto a nitrocellulose membrane, and probed with specific antibodies. Beta-actin was used as a loading control. The results are representatives of three independent experiments. **c.** Immunofluorescent staining of podoplanin (red) and phospho-E/R/M (green) proteins in BcPAP (right panels) and TPC1 cells (left panels) transfected with siPDPN or control siNeg. Cells were co-stained with the anti-PDPN mouse antibody D2–40 and anti-phospho-E/R/M rabbit antibody followed by the DyLight549-conjugated anti-mouse secondary antibody (red) and FITC-conjugated anti-rabbit secondary antibody (green), and counterstained with DAPI (blue). Magnification: 630x

There were no statistically significant differences in the E/R/M mRNA expression levels between PDPN-deprived and control cells (Fig. 4a). However, Western blot analyses showed increased levels of phospho-E/R/M proteins in both TPC1 and BcPAP cells after PDPN silencing compared to control cells transfected with siNeg (Fig. 4b). Immunofluorescence co-staining of PDPN and phospho-E/R/M confirmed that PDPN depleted cells, both TPC1 and BcPAP, expressed higher levels of phospho-E/R/M than control cells (Fig. 4c). The results indicate that a significant increase in the expression levels of phospho-E/R/M proteins is associated with the cellular expression of PDPN.

Furthermore, we analyzed the expression of E/R/M genes and proteins in response to PDPN silencing. We found that mRNA expression (Fig. 4a) and total E/R/M protein levels (Fig. 4b) did not change following siRNA transfection, whereas the levels of their phosphorylated forms increased.

### Cells phenotype and cytoskeleton organization changes

As PDPN was shown to mediate remodeling of the actin cytoskeleton and induce cell migration, we analyzed filamentous actin distribution and cell morphology of papillary thyroid cancer cells 48 h after PDPN silencing. For cytoskeleton visualization, the cells transfected with siPDPN or siNeg were grown and fixed on glass coverslips and then stained with Phalloidin-FITC which selectively labels F-actin.

**Fig. 5:**
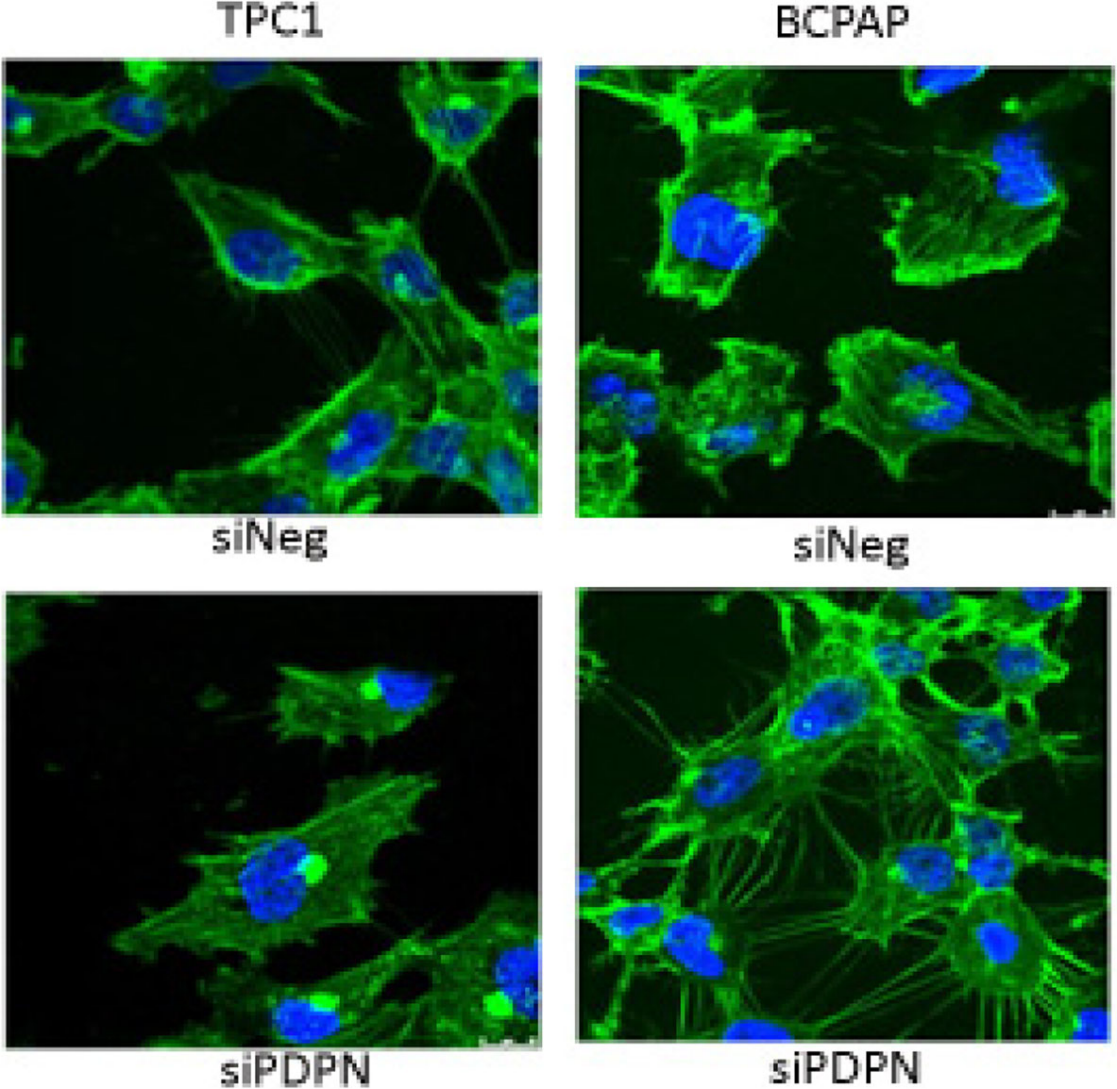
Silencing of PDPN changes cytoskeleton organization in TPC1 and BcPAP cells. Immunofluorescent staining of cytoskeleton in TPC1 and BcPAP cells transfected with siPDPN or control siNeg. To visualize filamentous actin (F-actin), cells were fixed with with 4% PFA/PBS, permeabilized with 0.1% Triton X-100, and stained with Phalloidin-FITC (green), and counterstained with DAPI (blue). Magnification: 630x

We observed that suppressing PDPN expression resulted in remodeling of cells’ shape and cytoskeleton organization in both TPC1 and BcPAP cells. However, the two cell lines had markedly different morphologies following PDPN silencing. In TPC1 cells, PDPN depletion resulted in the impaired cell spreading with reduced protrusions, in BcPAP cells it induced an increase in the number of cellular protrusions (Fig. 5).

### Podoplanin regulates the E/R/M and EMT pathways in thyroid cancer cells

**Fig. 6:**
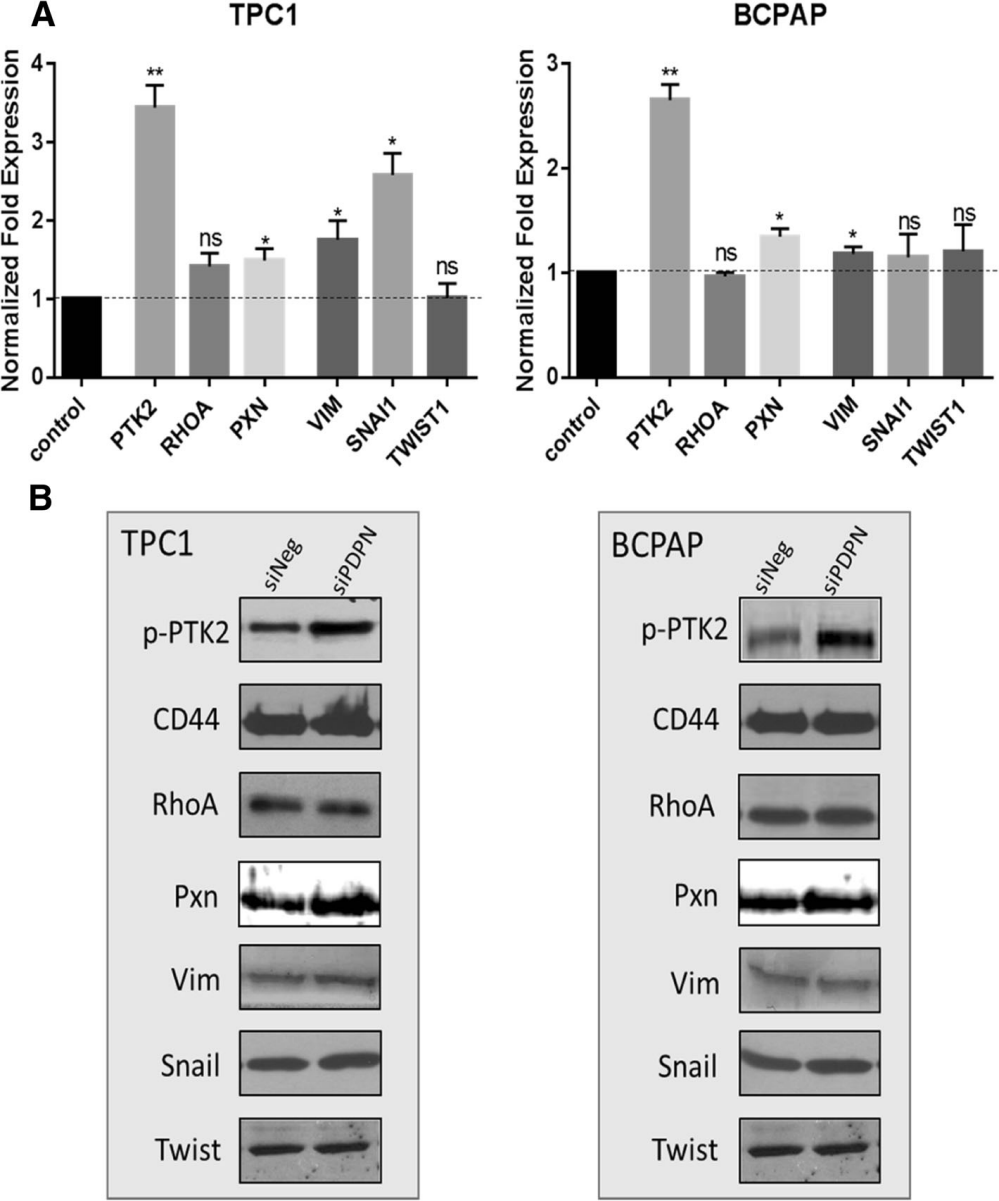
Podoplanin silencing does not alter the expression of proteins involved in the epithelial-mesenchymal transition (EMT) in thyroid cancer cells. **a.** RT-qPCR analysis of mRNA levels in TPC1 and BcPAP thyroid cancer cells 48 h after transfection. The results were normalized to the 18S rRNA levels. The bars represent the average fold change in transcripts levels in cells transfected with siPDPN compared to cells transfected with siNeg. Values are means from at least three independent experiments. Data are presented as means ± standard errors of the mean (SEM) *P < 0.05; **P < 0.01. **b.** Western blot analysis of CD44, RhoA, Twist, Snail, Vimentin, PTK2 expression levels 48 h after transfection with siPDPN or control siNeg in TPC1 and BcPAP cells. Total protein were separated by SDS-PAGE, transferred onto a nitrocellulose membrane, and probed with specific antibodies. The results are representatives of three independent experiments

In an attempt to elucidate the mechanism(s) through which PDPN affects the EMT pathway in papillary thyroid cancer, we studied the expression of several proteins involved in the E/R/M and EMT pathways following PDPN silencing in TPC1 and BcPAP thyroid cancer cell lines, i.e. CD44, RhoA, Snail, Twist1, Vimentin, Paxillin and PTK2. We found that PDPN knock-down did not change the expression levels of E/R/M regulators RhoA and CD44, nor that of Twist1, Vimentin, Paxillin and Snai1, in either of the studied cell lines (Fig. 6). Interestingly, we observed an enhanced expression and phosphorylation of protein tyrosine kinase 2 (PTK2; Fig. 6) which has been shown to play an important role in focal cellular adhesion.

### Podoplanin knock-down alters the expression and activity of MMPs in both BCPAP and TPC1 cell lines

**Fig. 7:**
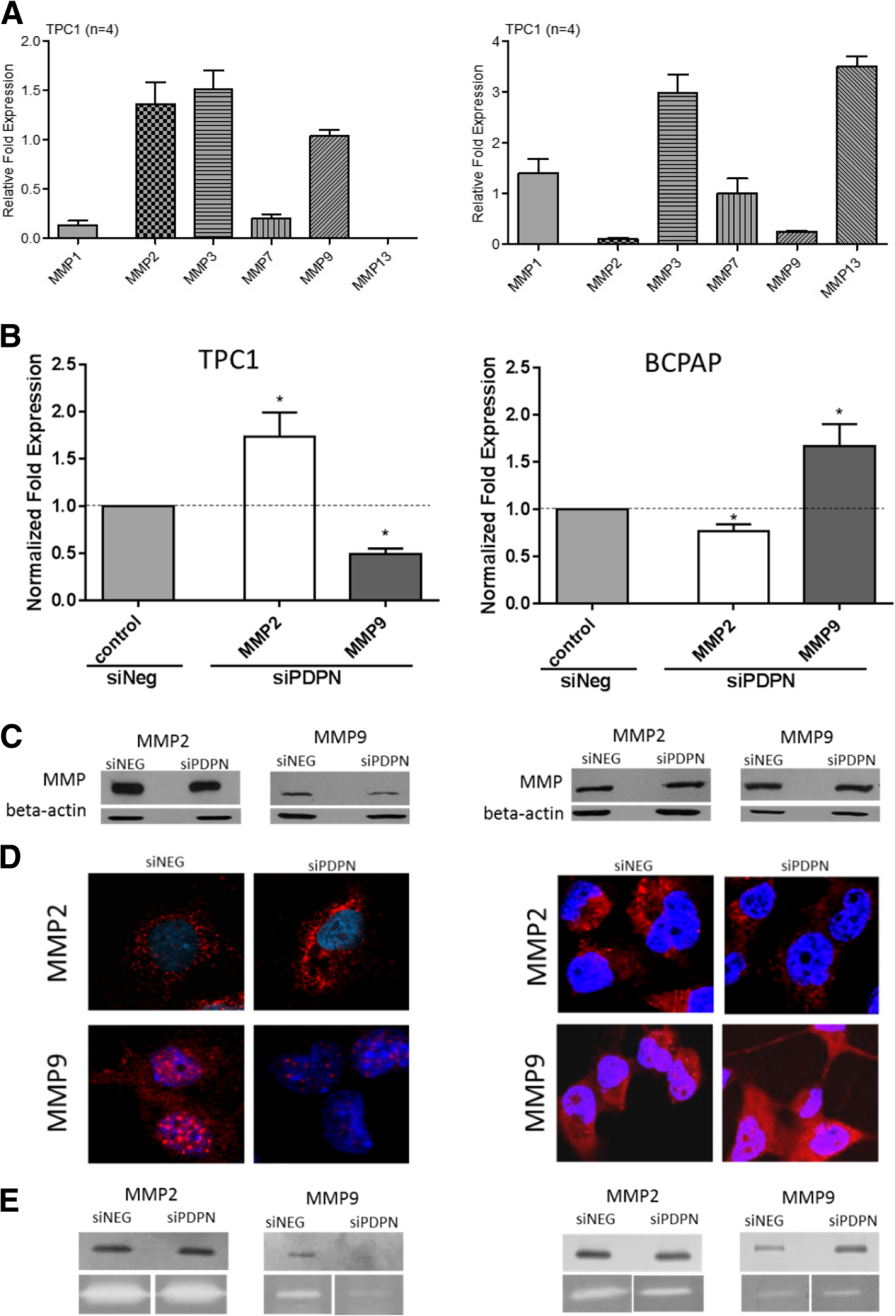
PDPN silencing alters the expression and activity of matrix metalloproteinases (MMPs) in TPC1 and BcPAP cells. **a.** The pattern of expression of MMPs mRNA levels in TPC1 and BcPAP cells. **b.** RT-qPCR analysis of MMP mRNA levels in 48 h after siPDPN transfection compared to those in non-transfected cells. The results were normalized to 18S RNA levels. The bars represent the average fold expression. Data are presented as means ± standard errors of the mean (SEM) of results from at least three independent experiments *P < 0.05**. c.** Western blot analysis of MMP expression levels 48 h after PDPN silencing. Total protein extracts were separated by SDS-PAGE, transferred onto a nitrocellulose membrane, and probed with specific antibodies. The results are representative of three independent experiments. **d.** Immunofluorescent staining for MMP-2 and MMP-9 in thyroid cancer cell lines transfected with siPDPN or control siNeg. Cells were fixed with 4% PFA/PBS, permeabilized with 0.1% Triton X-100, and stained with an anti-MMP-2 or anti-MMP-9 monoclonal antibody followed by the DyLight549-conjugated secondary antibody (red), and counterstained with DAPI (blue). Confocal microscopy magnification: 630x. **e.** Gelatin and collagen zymography for TPC1 and BcPAP cell lines after PDPN silencing

Since the invasion and metastasis of cells expressing PDPN seem to depend on the activity of matrix metalloproteinases, we analyzed the expression and activity of MMP-2 and MMP-9 in PDPN depleted TPC1 and BcPAP cell lines using RT-qPCR, immunofluorescence, and gelatin or collagen zymography. The two cell lines showed distinct profiles of MMP expression after PDPN silencing (Fig. 7a). We found that changes in the MMP expression after PDPN silencing were paralleled by alterations in the activity of MMPs as measured by zymography in both cell lines. Surprisingly, TPC1 and BcPAP cells displayed opposed patterns of MMP-2 and MMP-9 expression and activity before and after PDPN silencing. PDPN knock-down in TPC1 cells led to an increased expression and activity of MMP-2, whereas the expression and activity of MMP-9 were reduced. In contrast, in BcPAP cells, PDPN silencing resulted in an increased MMP-9 expression and activity, and decreased expression and activity of MMP-2 (Fig. 7b-e).

### Role of the MAPK signaling pathway

**Fig. 8:**
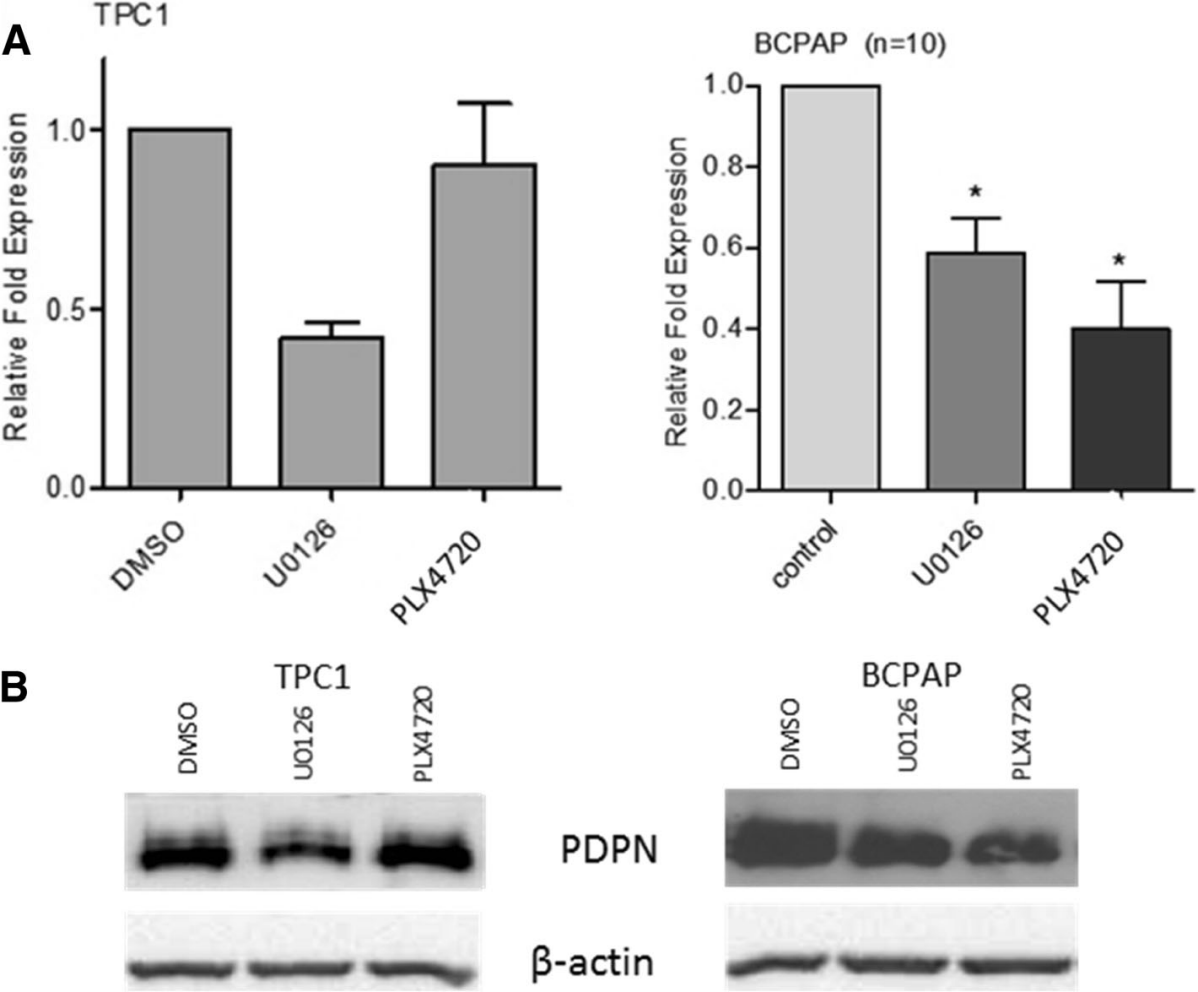
Effect of MAPK pathway inhibition on PDPN expression in BcPAP and TPC1 cell lines. **a.** RT-qPCR analysis of PDPN mRNA levels in BcPAP and TPC1 cells 24 h after treatment with 10 μM PLX4720 (*BRAF V600E* inhibitor), 50 μM U0126 (MEK1/2 inhibitor), or DMSO as a negative control. The results were normalized to 18S rRNA levels. The bars represent the average fold change in transcript quantity in cells treated with the inhibitors compared to cells treated with DMSO. The results are representative of three independent experiments. Data are presented as the means ± standard errors of the mean (SEM) * < P,0.05; ** < P,0.01. **b.** Western blot analysis of PDPN, phosphorylated ERK and β-actin protein levels in TPC1 and BcPAP cells 24 h after treatment with 10 μM PLX4720, 50 μM U0126, or DMSO as negative control

We hypothesized that one of the possible mechanisms responsible for observed PDPN-dependent phenotype in BcPAP cells, so different from that observed in TPC1, may be a constitutive activation of the BRAF pathway and downstream mitogen-activated protein kinase (MAPK) signaling. To test this hypothesis we inhibited the activity of the BRAF V600E or Mek1/2 allele using the PLX4720 or U0126 inhibitory compound, respectively. We observed that the inhibition of the MAPK pathway resulted in decreased levels of PDPN expression in both studied thyroid cancer cell lines. Consistently with the specificity of the used inhibitors, the PDPN transcript levels in BcPAP cells decreased by 60% after treatment with PLX4720 and by 40% when using UO126 (*P*-value < 0.05; Fig. 8a). In TPC1 cells, PDPN mRNA levels decreased by about 50% only after UO126 treatment (*P*-value < 0.01, Fig. 8a). Western blot analyses confirmed an efficient inhibition of the MAPK signaling by U0126 in TPC1 cells and by both U0126 and PLX47020 in BcPAP cells, as well as decreased PDPN expression following the treatment with the two inhibitors in both cell lines (Fig. 8b).

## Discussion

The expression of podoplanin (PDPN) has been found in various human cancers, including differentiated thyroid carcinoma. However, its role in carcinogenesis is still unclear. Many reports have suggested that it has a pro-metastatic activity [11, 18, 22, 24, 41]. In our previous study, we have shown that it is expressed only in PTC- derived cell lines (TPC1 and BcPAP, with higher expression levels in the latter cell line) and also in a proportion of human PTC tissues. The functional analysis of PDPN in TPC1 cells suggested that it has a pro-metastatic activity also in thyroid cancer, enhancing invasiveness of malignant thyroid cells [19]. To further assess the role of PDPN in the biology of papillary thyroid cancer, in the current study we assessed the effect of PDPN silencing in these two papillary thyroid cancer cell lines in more detail. Of note, these cell lines differ in their genetic background TPC1 harbors the *RET/PTC1* rearrangement, and BcPAP cell line carries the *BRAF V600E* mutation.

We had previously found that PDPN depletion in TPC1 cells significantly suppressed their in vitro motility, migration and invasion capacities, while cell viability, proliferation and apoptosis were not changed [19]. In this study PDPN depletion in these cells also affected anchorage–dependent and independent growth. Surprisingly, in BcPAP cells the PDPN depletion significantly increased their capacity to migrate and invade without detectable changes in viability, proliferation and apoptosis or cell cycle arrest. Moreover, reduced levels of PDPN correlated with an increased capacity for anchorage-independent growth and clonogenic outgrowth in these cells. Our results revealed an opposed effect of PDPN expression on malignant features of TPC1 and BcPAP cell lines. These two cell lines have different genetic background, that is either the *RET/PTC* translocation (TPC1), or *BRAF V600E* mutation (BcPAP). Therefore, the differences in the PDPN-induced phenotype could be related to the differences in their genetic background but further experiments are needed to test this hypothesis. We also found that modifying PDPN expression in both TPC1 and BcPAP cells did not affect the cell proliferation, apoptosis or cell cycle arrest. This suggests that PDPN activity is rather not dependent on the genetic background determining the regulation of these processes in papillary thyroid cancer cells.

It had been shown before that PDPN directly binds to ezrin and moesin and that its overexpression results in increased phosphorylation of the ezrin/radixin/moesin (E/R/M) proteins [11, 25]. E/R/M phosphorylation turns the proteins to active conformations that expose the binding sites which connect molecular complexes containing phospholipids, membrane receptors, transmembrane proteins to the actin filaments, thus playing a role in the regulation of the cytoskeleton and various cellular signaling pathways, cells motility and morphology [42, 43]. Therefore, we have analyzed the expression of the E/R/M mRNA and proteins as well as their phosphorylation status in the two thyroid cancer cell lines transfected with siPDPN and control siNeg. Surprisingly, the levels of phosphor-ERM increased markedly after PDPN depletion in both studied cell lines compared to control cells that had been transfected with siNeg, whereas both E/R/M transcript and protein level were not altered by transfection. Immunofluorescence co-staining of PDPN and phospho-E/R/M corroborated these results in both cell lines (Fig. 4c).

As PDPN was shown to mediate remodeling of the actin cytoskeleton and filopodia-like formation in addition to induction of cell migration [11, 44], we examined filamentous actin distribution and cell morphology of papillary thyroid cancer cells 48 h after PDPN silencing. We observed that suppressing PDPN expression significantly modified cytoskeleton and resulted in remodeling of cell shape in both TPC1 and BcPAP cells. Moreover, the morphology of cells from the two cell lines markedly differed. In TPC1 cells, PDPN depletion led to impaired cell-spreading with reduced protrusions, while in BcPAP cells PDPN knock-down induced an increase in the number of cellular protrusions. The observed morphological alterations were supported by the results of Western blot analyses which showed that expression levels of active forms of the E/R/M proteins increased with PDPN overexpression in papillary thyroid carcinoma cells. However, these observations do not explain the differences in response to the silencing of PDPN observed between the two cell lines. Therefore, we seeked to elucidate the underlying mechanisms of PDPN impact on the E/R/M and EMT pathways in papillary thyroid cancer by analyzing the main actors of these pathways.

Epithelial to mesenchymal transition (EMT) not only plays an important role during embryonic development but also contributes to pathological processes, such as invasion and metastatic dissemination of cancer cells [45, 46]. Among a variety of factors that promote EMT, the involvement of PDPN and its cell-type specific function in the EMT process, as well as the role of other regulatory proteins associated and/or activated with the process were also demonstrated in vitro [11, 25, 42] Therefore, we studied the expression of CD44, RhoA, Snail, Twist1, Vimentin, Paxillin and PTK2 proteins involved in the E/R/M or EMT pathway in TPC1 and BcPAP thyroid cell lines knock-down, which suggests that alternative signaling pathways were responsible for the observed changes in the cell motility and morphology. Interestingly, we detected enhanced expression and phosphorylation of protein tyrosine kinase 2 (PTK2; Fig. 6b) which has been shown to play an important role in focal cellular adhesion.

The invasion of tissues by cancer cells depends on the degradation of extracellular matrix (ECM) component and of the basement membrane surrounding the tumor. This complex multifactorial process involves several matrix metalloproteinases, including MMP-2 and MMP-9 that degrade ECM [21, 23, 24]. In our study, MMP-2 and MMP-9 in PDPN depleted TPC1 and BcPAP cells displayed inverse expression and activity patterns. In PDPN depleted TPC1 cells, we found a significant decrease in the MMP-9 transcript and protein expression, and its activity corroborated by immunofluorescence with MMP-2 expression not changed, whereas PDPN depleted BcPAP cells showed a significantly enhanced MMP-9 expression on both transcript and protein levels, confirmed by immunofluorescence and zymography. Parental TPC1 and BcPAP cells differ in the expression of MMP-2 and MMP-9, with BcPAP displaying very low expression of both MMPs analyzed, hence the differences in MMP-2 and MMP-9 expression following PDPN silencing in the two cell lines suggest that they may play a role in cancer progression, with different MMPs being involved, depending on the type of cells.

These results are in accordance with those previously reported by others. Studies on the PDPN function in cultured cancer cells, in mouse cancer models and in human cancer biopsies have indicated that PDPN promotes cells invasion in vitro and in vivo. Moreover, cellular invasion was totally abrogated after treatment of cultured cells with TIMP2, a natural inhibitor of MMPs [11]. Therefore, it seems that the invasive properties of PDPN-expressing cells depend on the activity of MMPs [11]. It was also shown that PDPN colocalizes with MMP-9 in the lymphatic endothelial cells [47]. The results of several studies have shown that PDPN is involved in ECM remodeling, and that the process of ECM degradation predominantly involves MMP-2 and MMP-9 whose expression correlates with prognosis in some cancer types [24, 47–52].

The characterstic genetic alterations harbored by the TPC1 and BcPAP activate the MAPK signaling pathway. To check whether there is an association between PDPN expression and the MAPK pathway activity, we inhibited components of the MAPK pathway: BRAFV600E and MEK1/2, in PDPN depleted cells. Interestingly, the inhibition of the MAPK pathway did not only decrease PDPN expression, but also increased phosphorylation of the E/R/M proteins and reduced cell migration in TPC1 cells, while in BcPAP cells, the inhibition of both BRAFV600E and MEK1/2 reduced cell motility. One of the possible mechanisms responsible for the observed PDPN phenotype in BcPAP cells, entirely different from that in the TPC1 cells, may be a constitutive activation of the BRAF pathway and downstream MAPK signaling. To test this hypothesis, we inhibited BRAFV600E and MEK1/2 separately. Our results showed that PDPN expression was associated with the MAPK signaling pathway since inhibited the mutated BRAF and the MEK1/2 kinase led to a reduction in PDPN protein expression. This is consistent with previous studies which had shown that the MAPK pathway was activated as a consequence of the *BRAF V600E* mutation, the mutant BRAF having a 500-fold greater kinase activity in vitro than the wild-type protein [53]. Consistently, PLX4720 effectively inhibited PDPN expression only in BcPAP cells, which carry mutated *BRAF*, and not in TPC1 cells.

Cell migration and invasion requires coordination between cytoskeletal reorganization, cell adhesion, interaction with ECM, and proteinase activity for ECM degradation. Our findings in thyroid cancer cells show that PDPN-induced changes of the tumor microenvironment promoted thyroid tumor cell motility, invasion, and metastasis. However, the role of PDPN was strongly dependent on the genetic background of the cells, and regulation of the MAPK pathway through E/R/M and MMPs activity is one of the possible mechanisms involved in these processes. PDPN may facilitate and increase the thyroid cell migration and invasion also by promoting cytoskeleton remodeling, which contributes to increased motility and invasion, and the association between PDPN expression and actin cytoskeleton seems to be mediated by the E/R/M proteins.

## Conclusions

We demonstrated that PDPN knock-down may promote or suppress metastatic potential of cells, depending on their genetic background. Moreover, our data suggest that PDPN pro-oncogenic function is correlated with the expression of E/R/M proteins and metalloproteinase (MMP) activity. We conclude that PDPN may play an important role in the regulation of invasion and migration of papillary thyroid carcinoma cells by activating E/R/M, MMPs and may also depend on MAPK kinase signaling. Further studies are needed to fully elucidate complex mechanisms underlying essential differences between papillary thyroid cancer cells with different genetic backgrounds and the role of podoplanin in acquiring metastatic potential.

## Additional file

Additional file 1: **Figure S1.** PDPN knock-down does not affect adhesiveness of TPC1 and BCPAP cells**.** A. Adhesion assay of TPC1 (upper graph) and BcPAP cells (lower graph) on 10 μg/ml fibronectin in 0.5; 1; 2 h after seeding. B. Adhesion assay of TPC1 (upper graph) and BcPAP cells (lower graph) on vitronectin, laminin, collagen and fibronectin. Error bars represent means ± standard errors of the mean (SEM) from three independent experiments. **Figure S2.** Effect of podoplanin silencing on cell cycle and viability of BcPAP and TPC1 cells. A. BrdU Cell Proliferation Assay of PDPN depleted and control cells. 48 h after siRNA transfection, the cells were incubated with BrdU for 17 h. Then, the cells were fixed with fixing solution, stained with anti-BrdU antibodies and incubated with TMB Peroxidase Substrate (blue). The intensity of the staining is proportional to the amount of BrdU incorporated by proliferating cells. Absorbance was measured at the test wavelength of 450 nm. Error bars represent means ± standard errors of the mean (SEM) from three independent experiments. B. Cell cycle assay. BcPAP and TPC1 cells after siPDPN or siNeg transfection and control cells (no siRNA) were fixed, permeabilized, stained with propidium iodide (PI) solution, and then analyzed by flow cytometry. The results are presented as percentage of cells in G1, S, and G2/M phases. (ZIP 907 kb)

## Abbreviations

BcPAP: Papillary thyroid carcinoma
BRAF: B-type Raf kinase
CD44: Cluster of differentiation 44
DMSO: Dimethyl sulfoxide
ERK: Extracellular-signal regulated kinase
RT-qPCR: quantitative real-time reverse transcription - polymerase chain reaction
MAPK: Mitogen activated protein kinase
MEK: Mitogen-activated protein kinase
MMPs: Matrix metalloproteinases
PTC: Papillary thyroid cancer
RET: Rearranged during transfection
RhoA: Ras homolog family member A
siRNA: Small interfering RNA
