# Supplementary figures and images for: Podoplanin (PDPN) affects the invasiveness of thyroid carcinoma cells by inducing ezrin, radixin and moesin (E/R/M) phosphorylation in association with matrix metalloproteinases
Source: BMC Cancer. 2019 Jan 17;19:85. doi: 10.1186/s12885-018-5239-z (PMC6337816; doi:10.1186/s12885-018-5239-z)

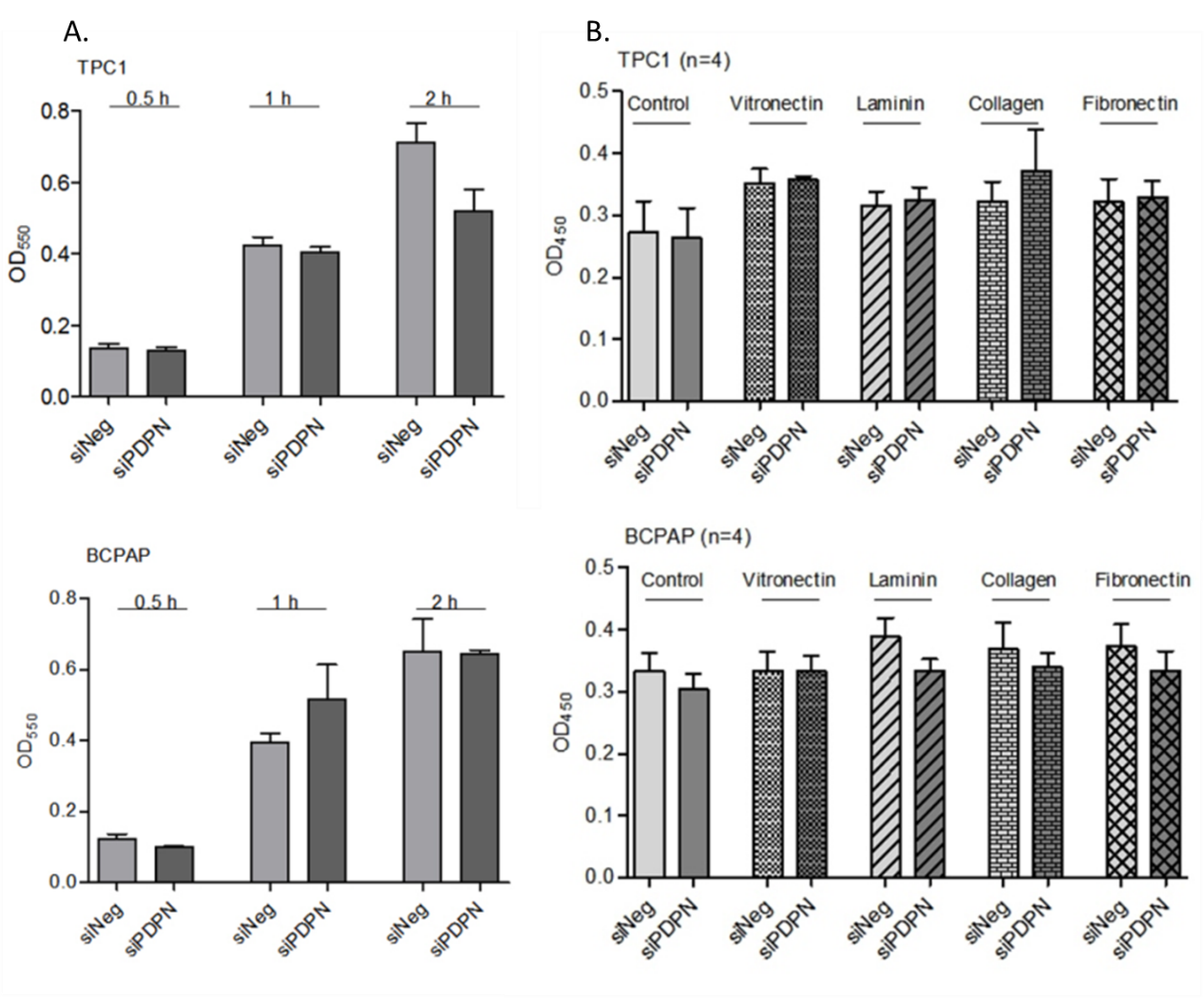

Supplement: Supplementary file 1 — Figure S1. PDPN knock-down does not affect adhesiveness of TPC1 and BCPAP cells. A. Adhesion assay of TPC1 (upper graph) and BcPAP cells (lower graph) on 10 μg/ml fibronectin in 0.5; 1; 2 h after seeding. B. Adhesion assay of TPC1 (upper graph) and BcPAP cells (lower graph) on vitronectin, laminin, collagen and fibronectin. Error bars represent means ± standard errors of the mean (SEM) from three independent experiments. Figure S2. Effect of podoplanin silencing on cell cycle and viability of BcPAP and TPC1 cells. A. BrdU Cell Proliferation Assay of PDPN depleted and control cells. 48 h after siRNA transfection, the cells were incubated with BrdU for 17 h. Then, the cells were fixed with fixing solution, stained with anti-BrdU antibodies and incubated with TMB Peroxidase Substrate (blue). The intensity of the staining is proportional to the amount of BrdU incorporated by proliferating cells. Absorbance was measured at the test wavelength of 450 nm. Error bars represent means ± standard errors of the mean (SEM) from three independent experiments. B. Cell cycle assay. BcPAP and TPC1 cells after siPDPN or siNeg transfection and control cells (no siRNA) were fixed, permeabilized, stained with propidium iodide (PI) solution, and then analyzed by flow cytometry. The results are presented as percentage of cells in G1, S, and G2/M phases. (ZIP 907 kb) [file 12885_2018_5239_MOESM1_ESM.zip › 12885_2018_5239_MOESM1_ESM.docx]

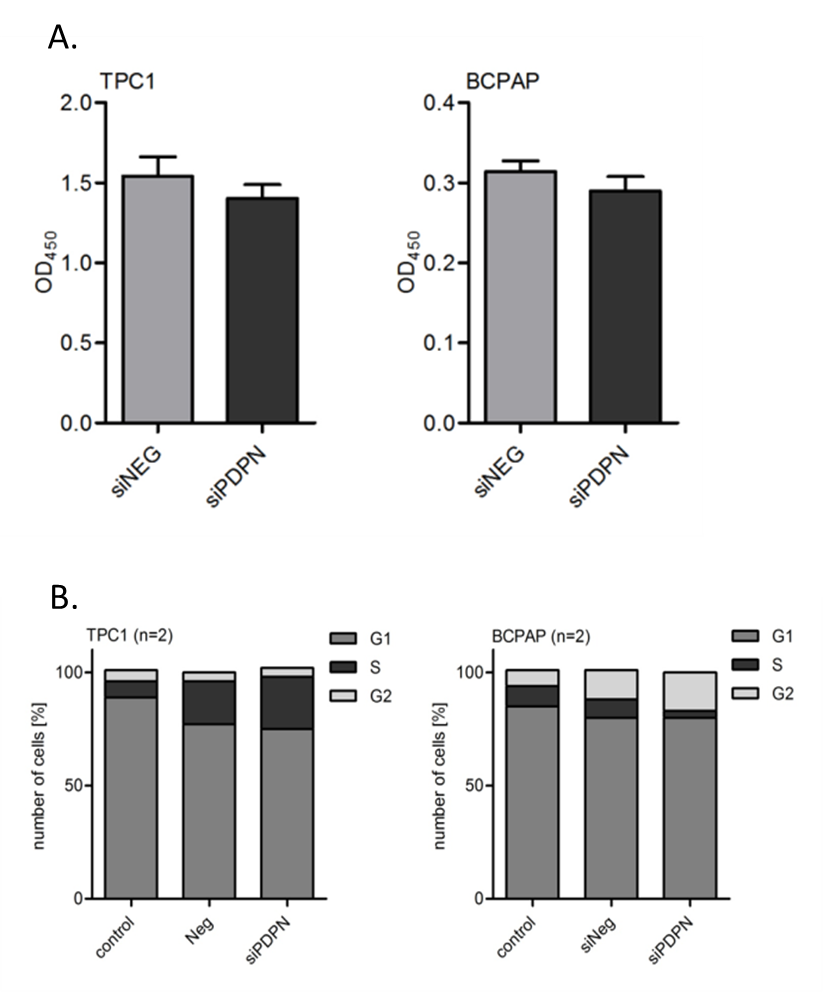

Supplement: Supplementary file 1 — Figure S1. PDPN knock-down does not affect adhesiveness of TPC1 and BCPAP cells. A. Adhesion assay of TPC1 (upper graph) and BcPAP cells (lower graph) on 10 μg/ml fibronectin in 0.5; 1; 2 h after seeding. B. Adhesion assay of TPC1 (upper graph) and BcPAP cells (lower graph) on vitronectin, laminin, collagen and fibronectin. Error bars represent means ± standard errors of the mean (SEM) from three independent experiments. Figure S2. Effect of podoplanin silencing on cell cycle and viability of BcPAP and TPC1 cells. A. BrdU Cell Proliferation Assay of PDPN depleted and control cells. 48 h after siRNA transfection, the cells were incubated with BrdU for 17 h. Then, the cells were fixed with fixing solution, stained with anti-BrdU antibodies and incubated with TMB Peroxidase Substrate (blue). The intensity of the staining is proportional to the amount of BrdU incorporated by proliferating cells. Absorbance was measured at the test wavelength of 450 nm. Error bars represent means ± standard errors of the mean (SEM) from three independent experiments. B. Cell cycle assay. BcPAP and TPC1 cells after siPDPN or siNeg transfection and control cells (no siRNA) were fixed, permeabilized, stained with propidium iodide (PI) solution, and then analyzed by flow cytometry. The results are presented as percentage of cells in G1, S, and G2/M phases. (ZIP 907 kb) [file 12885_2018_5239_MOESM1_ESM.zip › 12885_2018_5239_MOESM2_ESM.docx]
